# Chromosome-level genome assembly of the sand martin (*Riparia riparia*)

**DOI:** 10.1038/s41597-026-07311-2

**Published:** 2026-05-07

**Authors:** Miroslav Nuriddinov, Lyubov Malinovskaya, Aleksandr Bobrovskikh, Maria Gridina, Natalya A. Serdyukova, Alexander Suh, Francisco J. Ruiz-Ruano, Anna Torgasheva

**Affiliations:** 1 Sirius University of Science and Technology, Sirius Federal Territory, Sochi, Russia; 2 Institute of Cytology and Genetics, Russian Academy of Sciences, Novosibirsk, Russia; 3 Novosibirsk State University, Novosibirsk, Russia; 4 Institute of Molecular and Cellular Biology, Russian Academy of Sciences, Novosibirsk, Russia; 5 Centre for Molecular Biodiversity Research, Leibniz Institute for the Analysis of Biodiversity Change, Museum Koenig Bonn, Bonn, Germany; 6 Bonn Institute for Organismic Biology – Animal Biodiversity, University of Bonn, Bonn, Germany

**Keywords:** Genome informatics, Sequence annotation

## Abstract

The sand martin (*Riparia riparia*), a widely distributed migratory songbird, is a promising model for evolutionary and population genetics due to its unique life-history traits. It also provides a valuable system for studying germline-restricted chromosome (GRC) inheritance and evolution. However, the absence of a high-quality genomic resource has limited in-depth investigation of these phenomena. Here, we present a chromosome-level somatic genome assembly for a *R. riparia* male generated using PacBio HiFi long-read sequencing and Hi-C scaffolding. The pseudohaploid assembly spans 1.19 Gb across 40 chromosome models and shows high completeness (97.6% BUSCO score). Repetitive elements make up 20.2% of the assembled chromosomes. A total of 19,624 protein-coding genes were annotated by integrating transcriptome evidence, *ab initio* gene prediction, and homology-based approaches. This high-quality reference genome provides a valuable foundation for studying population structure, adaptation, and evolutionary history in *R. riparia*. It serves as a critical resource for future assembly and investigation of the GRC, and contributes to a broader understanding of genome evolution in birds.

## Background & Summary

The sand martin (*Riparia riparia*), commonly known as the bank swallow, is widely distributed across a vast geographical range. These migratory birds exhibit remarkable adaptability to various environmental conditions, contributing to their substantial population size^1^. Combined with their biological traits - such as monogamy, philopatry, and a large number of offspring - this makes them a unique model for evolutionary genomics. Although previous studies based on limited genetic markers suggested shallow population structure and recent demographic expansion^2–5^, and a linked-read draft genome assembly has been published^6^, the lack of a chromosome-level reference genome assembly has so far limited deeper insights into the sand martin’s evolutionary history and adaptive potential.

As a widely distributed migratory colonial species classified as Least Concern (LC), the sand martin offers a valuable system for studying the inheritance, population genetics, and evolution of the germline-restricted chromosome (GRC)^2,7^. Recent comparative genomic analysis suggests that GRCs are likely present in two-thirds of all bird species (~6,700 passerines), evolving rapidly while retaining functionally important genes^7^. However, its origin, function, and mechanisms of inheritance remain unclear. Sand martins possess a macro-GRC characterized by mosaicism and polymorphism^3^. It contains sequences homologous to those in the standard chromosomal set (A chromosomes), some of which are present in multiple copies^3^. Due to their highly repetitive nature, macro-GRCs cannot be assembled to A-chromosome-level quality, even when combining HiFi and ONT reads, as demonstrated by a recent comprehensive analysis of the zebra finch GRC^7^. Obtaining a reliable GRC assembly in sand martin critically depends on the availability of a high-quality somatic genome assembly with gene annotation.

**Fig. 1:**
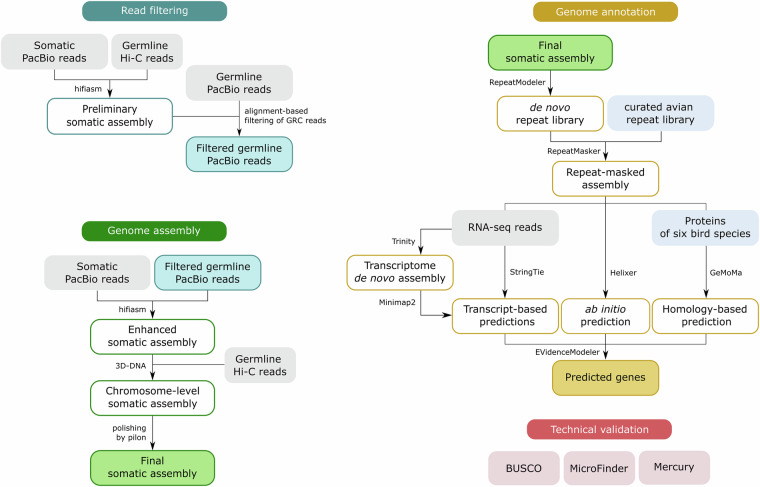
Workflow for generating a chromosome-level assembly and annotation of the sand martin genome.

In this study, we present a chromosome-level, pseudohaploid somatic genome assembly of a male sand martin using PacBio HiFi long-read sequencing and Hi-C technologies (Fig. 1). The final assembly spans 1.19 Gb, with a scaffold N50 of 73.3 Mb across 40 chromosome models, matching the known somatic karyotype of the species (2n = 40). The assembly includes well-represented dot chromosomes (16 and 30–37), which are typically missing or highly fragmented in avian genome assemblies. These chromosomes comprise 7.1% of the genome and show high gene content completeness. We annotated 19,624 genes, comprising a total of 185,064 exons, by integrating transcriptomic, *ab initio*, and homology-based predictions. This reference genome provides a valuable resource for evolutionary and functional genomic studies in birds and lays a foundation for future assembly and investigation of the structure and function of the GRC in sand martin.

## Methods

### Sample collection and sequencing

Two adult males and three adult females of sand martin were captured using bird nets at the end of the breeding season (mid-June 2023) near Novosibirsk (54.958348°N, 83.172235°E). Capture, handling, and euthanasia of the birds followed the protocols approved by the Ethics Committee on Animal Care and Use of the Institute of Cytology and Genetics (approval No. 179 of 26 August, 2024). Experiments described in this manuscript were carried out in accordance with the approved national guidelines for the care and use of animals.

To assemble the genome, we used PacBio HiFi sequencing data from somatic (muscles) and germline (testes) tissues of sand martin male #1. The choice of the tissues was motivated by our interest in the subsequent identification of GRC-specific sequences, which is beyond the scope of this work.

Genomic DNA from muscles and one testis of male #1 was extracted using the standard phenol-chloroform method. The integrity of the extracted DNA was assessed using 0.5% agarose gel electrophoresis. The DNA library was prepared by BGI and sequenced on the PacBio Revio long-read sequencing platform. For the muscles, we obtained 2,876,869 raw reads with a total size of 39.47 Gb (N50 = 13,717 bp with an average read length of 13,721 bp and a mean read quality of 32.0). For the testis, we obtained 4,693,444 raw reads with a total size of 72.34 Gb (N50 = 15,419 bp with an average read length of 15,413 bp and a mean read quality of 30.8) (Table 1).

**Table 1:** Statistics of the sequencing data used for sand martin genome assembly.

Tissue	Strategy	Platform	Raw data (Gb)	N reads	Coverage (X)
Muscle (male #1)	Long-read	PacBio HiFi Revio	39.47	2,876,869	~35
Testis (male #1)	Long-read	PacBio HiFi Revio	72.34	4,693,444	~60
Testis (male #1)	Hi-C	DNBseq-G400	121.91	406,379,160	~100
Mix of somatic tissues (male #2)	RNA-seq	DNBseq™	20.07	133,773,050	—
Mix of somatic tissues (females #1–3)	RNA-seq	DNBseq™, NovaSeq X Plus	64.48	563,603,298	—

The second testis of male #1 was used for Hi-C library preparation. The Hi-C library was prepared according to Belaghzal *et al*.^8^ using DpnII to fragment DNA and KAPA HyperPlus kit (Basel, Switzerland) as per the manufacturers’ protocol. The Hi-C library was sequenced by the BGI company on the DNBSEQ platform with a paired-end 150 bp layout, producing a total of 121.91 Gb of Hi-C read data.

For transcriptome and genome annotation, we performed mRNA sequencing of somatic tissues (kidneys, lungs, and muscles) from male #2 and three females (#1–3). Total RNA was extracted using Aurum Total RNA Mini Kit (Bio-Rad, USA) according to the manufacturer’s instructions. RNA quality was assessed using a NanoDrop 2000 spectrophotometer (Thermo Fisher Scientific, USA). Strand-specific mRNA libraries were constructed from poly(A)-enriched RNA and sequenced by the BGI on the DNBSEQ sequencing platform (male #2 and female #1) and by the Novogene on the NovaSeq X Plus sequencing platform (females #2 and #3) with a paired-end 150 bp layout, resulting in 20.07 Gb of data for the male and 64.48 Gb for females.

### Genome assembly

To assemble the somatic genome, we combined PacBio long-read sequencing data from somatic and germline tissue of the same specimen. In order to use sequencing data from germline tissue for somatic genome assembly, we filtered out most GRC-specific sequences as follows (Fig. 1).

First, we assembled somatic PacBio HiFi reads with hifiasm (v0.19.9-r616, default parameters), using Hi-C reads as long-range data^9^. The assembly yielded 2,687 contigs with a total length of 1,398.63 Mb for both haplotypes (Table 2). This preliminary assembly was used exclusively as a reference for aligning and filtering germline reads.

**Table 2:** Summary statistics of intermediate sand martin genome assemblies.

Assembly	Input data	Haplotype	Total length (bp)	Number of scaffolds	Scaffold N50 (bp)	L50
Preliminary somatic assembly used for germline read filtering	Somatic PacBio reads; germline Hi-C reads	haplotype 1	1,248,568,877	2,809	2,793,893	101
		haplotype 2	1,353,407,289	2,348	2,733,925	118
		both haplotypes	1,398,630,185	2,687	6,264,242	54
Enhanced somatic assembly used for downstream analyses	Somatic PacBio reads; filtered germline PacBio reads	haplotype 1	1,402,073,485	2,570	4,322,616	73
		haplotype 2	1,293,826,545	1,795	4,005,491	73
		both haplotypes	1,421,797,909	2,023	11,382,440	30

Using LastZ v1.04.22^10^, we aligned germline reads to both haplotypes of the preliminary somatic assembly. We filtered out reads with less than 99% sequence identity and aligned over less than 50% of their length, which yielded 3,659,752 reads (78% of the total reads from testis). The GRC length was estimated through cytological analysis to be approximately 10% of the total genome length^3^. Given this and the fact that the GRC is present only in a minority of testicular cells (spermatogonia and primary spermatocytes^3^), we suppose that these filtering criteria were sufficiently stringent to remove most GRC-specific reads.

Then, we combined the filtered germline and somatic reads (6,536,621 reads, 95.94 Gb, ~80× coverage) to generate a new haplotype-resolved diploid assembly using hifiasm (v0.19.9-r616, default parameters) without incorporating Hi-C data. The resulting assembly comprised 2,023 contigs (Table 2).

To improve the assembly using Hi-C contact pattern information, we aligned Hi-C reads to both haplotypes of the resulting contig-level assembly with Juicer v1.6^11^ and scaffolded with 3D-DNA v180922^12^ (with zero rounds of polishing). We manually curated 3D-DNA scaffolds to obtain a chromosome-level assembly. To assign scaffolds to chromosomes, we aligned the zebra finch (*Taeniopygia guttata*) somatic reference genome (bTaeGut1.4.pri, GenBank accession GCA_003957565.4)^13^ to our assembly using LastZ with the following parameters: --hspthresh = 3000 --gfextend --chain --gapped --matchcount = 100. In cases where the Hi-C contact pattern conflicted with the alignment-based assignment, scaffolds were grouped into chromosomes according to the Hi-C contact maps.

**Fig. 2:**
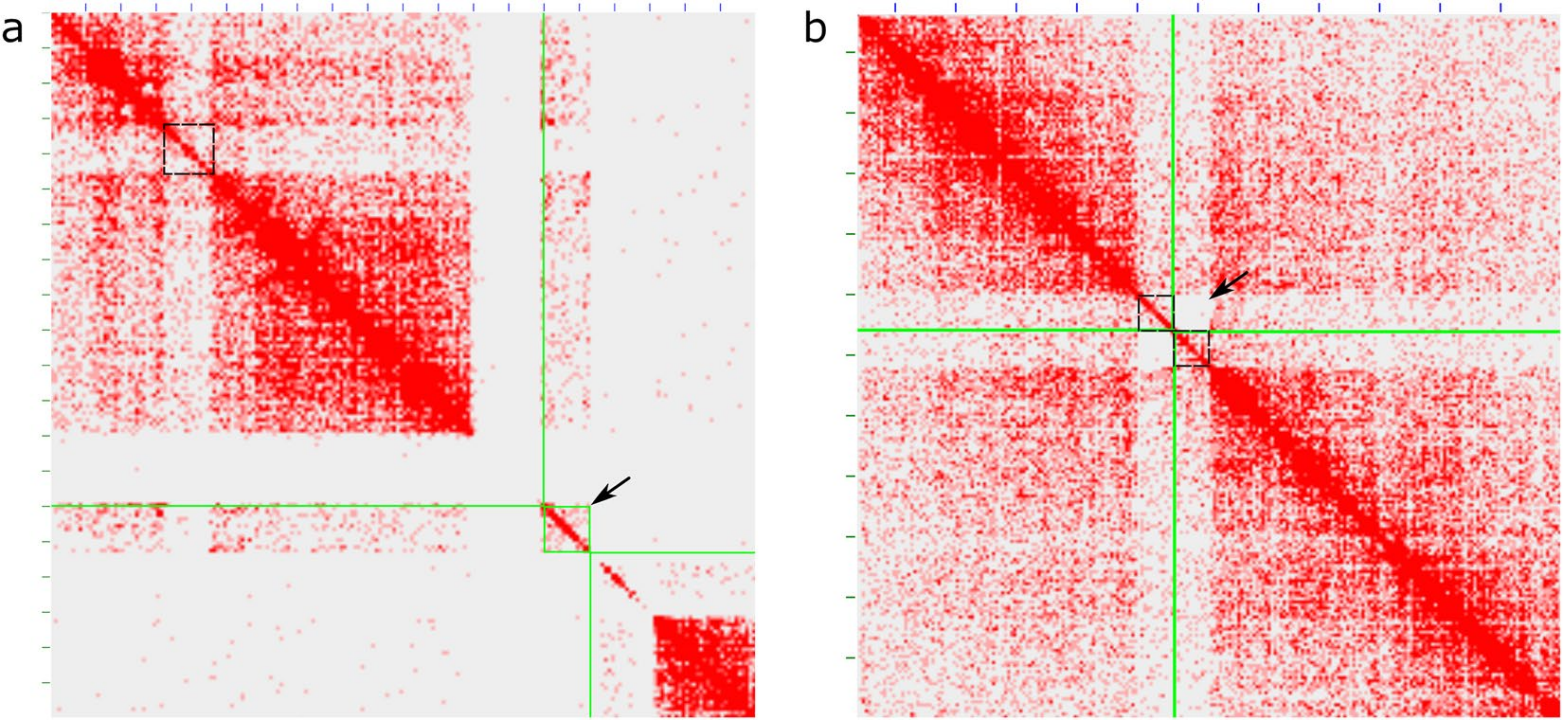
Examples of haplotype resolution through manual curation of the Hi-C contact map scaffolded and visualized with 3D-DNA. (**a**) A short contig (arrow) duplicates a fragment (black square) of a longer one and was considered a haplotypic copy. (**b**) Parts of two contigs (black squares) exhibit identical Hi-C contact profiles but show reduced Hi-C contact frequency between them (arrow), suggesting haplotypic origin. Green lines indicate the contig boundaries. Axis tick spacing: 50 (a) and 100 (b) kb.

We manually resolved duplicated regions corresponding to alternative haplotypes using Hi-C pattern. Two common patterns of duplication are shown in Fig. 2. Candidate regions were confirmed by aligning contigs with identical Hi-C contact patterns using LastZ v1.04.22^10^ with the following parameters: --hspthresh = 6000 --gfextend --chain --gapped --matchcount = 1000. We considered a locus haplotype-specific when over 80% of its sequence was unique and did not overlap with the aligned region on the matching scaffold. Based on this alignment, we identified haplotype boundaries at single-nucleotide resolution. Using these criteria, we removed redundant haplotypic sequences and generated a primary, pseudohaploid assembly approximating a single haplotype.

To minimize the potential impact of GRC-derived polymorphisms, we performed four rounds of polishing the assembly with Pilon v1.24^14^ (--fix gaps,local --diploid) using PacBio somatic reads. Using BEDTools^15^, we identified regions not covered by PacBio somatic reads and excluded them from the assembly. The remaining assembly was then polished twice more using somatic reads. The final assembly had a total length of 1.189 Gb with a scaffold N50 of 73.3 Mb and 40 chromosomes (Table 3). This outcome aligns with the known somatic karyotype of sand martin (2n = 40)^3^. The size of the assembly is consistent with the publicly available pseudohaploid linked-read draft genome assembly of sand martin (1.18 Gb)^6^. However, a Feulgen image analysis densitometry estimate suggests a slightly larger haploid genome size for somatic tissue (C-value = 1.45 pg, ≈1.42 Gb)^16^.

**Table 3:** Chromosome-level genome assembly statistics for the sand martin.

Chromosome	Length (bp)	Portion of genome	# contigs	% GC
1	123,619,578	10.39%	10	40.30%
1 A	78,596,128	6.61%	14	40.75%
2	162,371,519	13.65%	29	39.99%
3	122,460,553	10.30%	23	40.43%
4	73,324,403	6.16%	5	39.89%
4 A	9,974991	0.84%	1	46.34%
5	65,349,720	5.49%	4	41.79%
6	37,375,236	3.14%	2	42.93%
7	39,341,738	3.31%	4	42.26%
8	31,612,317	2.66%	2	43.09%
9	25,862,010	2.17%	1	44.06%
10	21,091,727	1.77%	8	44.34%
11	22,713,200	1.91%	2	44.07%
12	21,557,546	1.81%	2	44.80%
13	19,431,818	1.63%	6	46.48%
14	17,214,483	1.45%	3	46.63%
15	14,505,811	1.22%	3	47.72%
16	12,850,964	1.08%	22	50.66%
17	11,733,768	0.99%	1	49.61%
18	12,838,195	1.08%	5	48.24%
19	12,712,807	1.07%	1	48.27%
20	15,654,453	1.32%	2	47.77%
21	8,283,370	0.70%	12	50.28%
22	6,695,316	0.56%	4	52.34%
23	9,766,432	0.82%	5	51.83%
24	9,277,976	0.78%	1	50.90%
25	4,309,749	0.36%	5	56.50%
26	7,741,433	0.65%	2	53.21%
27	6,350,061	0.53%	3	53.87%
28	7,770,349	0.65%	3	53.86%
29	7,224,433	0.61%	12	52.92%
30	4,942,280	0.42%	17	53.76%
31	9,693,878	0.82%	33	52.50%
32	4,473,711	0.38%	11	49.30%
33	12,666,737	1.06%	13	51.03%
34	8,952,338	0.75%	15	51.48%
35	7,575,968	0.64%	19	51.09%
36	18,749,177	1.58%	37	48.61%
37	4,769,738	0.40%	25	51.97%
Z	97,956,271	8.24%	32	40.91%
Total	1,189,392,182	100%	399	43.07%

**Fig. 3:**
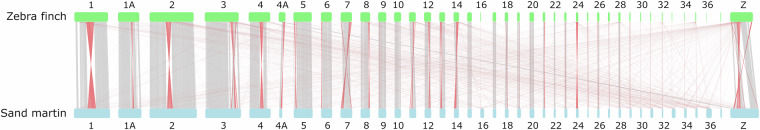
Whole-genome alignment of zebra finch and sand martin genome assemblies at nucleotide levels. Gray connections indicate direct alignments, while red connections indicate inverted alignments.

To identify synteny and structural rearrangements, we aligned the sand martin assembly to the zebra finch somatic reference genome (bTaeGut1.4.pri, GenBank accession GCA_003957565.4) using LastZ^10^ with the following parameters: high-scoring segment pairs (HSPs) threshold (-hspthresh) = 6000, interpolation threshold (-inner) = 2000, step size (-step) = 20, alignment processed with gap-free extension of seeds, gaps extension of HSPs and excluded chaining if HSPs (-gfextend -nochain -gapped). This analysis revealed a high degree of synteny consistent with the generally low frequency of interchromosomal rearrangements in avian genomes^17^ (Fig. 3). The translocation of a ~10 Mb region from chromosome 4A to the Z chromosome is in agreement with previously reported rearrangements observed across all studied species of Sylvioidea^18,19^.

### Transcriptome assembly

We assembled the transcriptome of sand martin using four somatic tissue samples (each a mixture of kidneys, lungs, and muscles) from one male and three females. For the primary transcriptome assembly, we used Trinity v2.15.2^20^ with the parameters --seqType fq --max _memory 500 G --CPU 16. This step yielded 744,217 potential transcript isoforms. To identify the most representative and reliable isoforms, we performed pseudo-alignment of transcriptome reads to the assembled isoforms using Salmon v1.10.3^21^. The resulting counts were imported using the tximport package v1.36.1^22^, combined into a single count matrix, normalized (calcNormFactors, method = “TMM”), and filtered (counts-per-million ≥ 1 in at least 2 of 6 libraries) using edgeR v4.4.2^23^.

This filtering step retained 61,309 transcripts. We extracted the longest isoform for each transcript using the get_longest_isoform_seq_per_trinity_gene.pl script provided by Trinity, resulting in a final set of 27,216 transcripts. To assess transcriptome completeness, we ran BUSCO v5.8.2^24^ using the Aves lineage dataset (odb10). The results showed 78% completeness, with 75.7% single-copy, 2.3% duplicated, and 2.4% fragmented orthologs. Given the limited range of sampled tissues, this transcriptome likely does not capture the full diversity of expressed genes, reinforcing the need to incorporate additional lines of evidence in genome annotation.

### Genome annotation

**Fig. 4:**
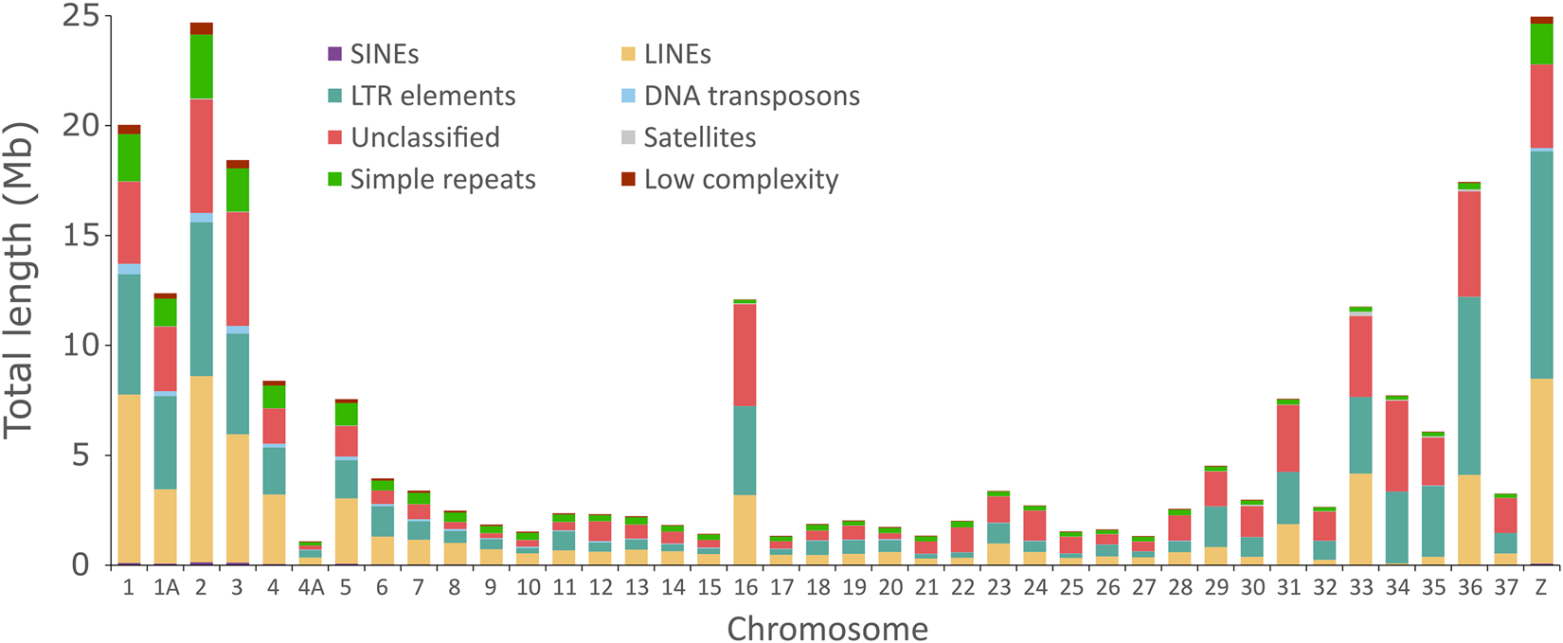
Chromosomal distribution of repeat classes in the sand martin genome assembly. Stacked bars indicate total repeat length (Mb) per chromosome.

To annotate repetitive elements, we generated a *de novo* repeat library using RepeatModeler v2.0.7^25^ with the -LTRStruct option, combined it with a curated avian repeat library from Peona *et al*.^26^, and soft-masked the assembly using RepeatMasker v4.2.3-p1^27^ with RMBlast v2.14.1 search engine. The repeat elements accounted for 20.2% (Table 4) of the genome predominated by LINEs (5.8%) and LTR elements (6.4%) (Fig. 4).

**Table 4:** Repetitive elements identified by RepeatMasker in the sand martin assembled chromosomes.

	Count	Length, bp	Percentage of sequence
***Retroelements***	276,469	146,533,430	12.32
** SINEs**	8,041	935,587	0.08
** Penelope**	0	0	0.00
** LINEs**	167,996	69,497,361	5.84
CRE/SLACS	0	0	0.00
L2/CR1/Rex	164,928	69,013,435	5.80
R1/LOA/Jockey	0	0	0.00
R2/R4/NeSL	2,203	369,103	0.03
RTE/Bov-B	21	1,516	0.00
L1/CIN4	69	15,629	0.00
** LTR elements**	100,432	76,100,482	6.40
BEL/Pao	840	813,514	0.07
Ty1/Copia	0	0	0.00
Gypsy/DIRS1	137	10,714	0.00
Retroviral	87,009	69,539,147	5.85
***DNA transposons***	17,721	2,892,412	0.24
hobo-Activator	2,503	427,378	0.04
Tc1-IS630-Pogo	693	194,560	0.02
En-Spm	0	0	0.00
MULE-MuDR	0	0	0.00
PiggyBac	183	28,693	0.00
Tourist/Harbinger	4,830	473,252	0.04
Other	0	0	0.00
***Rolling-circles***	212	23,244	0.00
***Unclassified***	96,125	66,070,720	5.55
***Small RNA***	2,183	280,198	0.02
***Satellites***	3,169	785,971	0.07
***Simple repeats***	379,392	20,474,975	1.72
***Low complexity***	61,493	3,663,184	0.31
**All repeats**	—	240,445,108	20.22

We annotated genes through a combination of three approaches: (1) transcript-based prediction, based on RNA-seq reads aligned with HISAT2 v2.2.1^28^ and processed using StringTie v.2.2.3^29^ with default options and *de novo* Trinity assembly mapped with minimap2 v.2.29-r1283^30^; (2) *ab initio* prediction from Helixer v0.3.4 (run in Vertebrate mode; https://www.plabipd.de/helixer_main.html)^31^; and (3) homology-based prediction generated by GeMoMa v.1.9^32,33^, which mapped protein sequences from six reference species: *Gallus gallus* (bGalGal1.mat.broiler.GRCg7b, GCF_016699485.2)^34^, *Serinus canaria* (serCan2020, GCF_022539315.1)^35^, *Passer domesticus* (bPasDom1.hap1, GCF_036417665.1), *Parus major* (Parus_major1.1, GCF_001522545.3)^36,37^, *T. guttata* (bTaeGut1.4.pri, GenBank accession GCA_003957565.4)^13^, and *Hirundo rustica* (bHirRus1.pri.v3, GCF_015227805.2)^38^ onto the assembly. These evidence sources were combined into a consensus gene set using EVidenceModeler v2.1.0^39^, with weights of 5, 3, 2, and 1 assigned to Trinity, GeMoMa, Helixer, and StringTie models, respectively. A total of 19,624 unique genes with 185,064 exons were identified. Gene length ranged from 150 to 198,566 bp, with a median length of 7578 bp. Exon counts per gene ranged from 1 to 201, with a median of 7.

## Data Records

Raw sequencing data generated in this study have been deposited in the NCBI database under accession number SRP610305^40^ (BioProject PRJNA1308776), including PacBio HiFi (accession number SRX30176648 for muscle and SRX30176649 for testis), Hi-C (SRX30176650), and RNA-seq reads (accession number SRX30176651 for male somatic tissues; SRX30176652, SRX30176653 and SRX30304808 for female somatic tissues). The final chromosome-level genome assembly is available from NCBI under accession number GCA_055001455.1^41^. The corresponding annotation files, including the masked genome assembly (riRi.soma-enhanced.both.rf_p2.masked.fa), *de novo* transcriptome assembly (trinity_expressed_longest_iso.fasta), repeat annotations (riRi.soma-enhanced.both.rf_p2.RepeatMasker.bed) with associated statistics (repeats.statistic.tsv), gene predictions based on homology (riRi_GeMoMa.gff3) and *ab initio* approaches (riRi_Helixer.gff3), RNA-seq–based gene models (riRi_StringTie.gff3), transcriptome-based gene models (riRi_Trinity.gff3), and the final consensus gene annotation (sandmartin.EVM.3.gff3), are available via Figshare^42^.

## Technical Validation

**Fig. 5:**
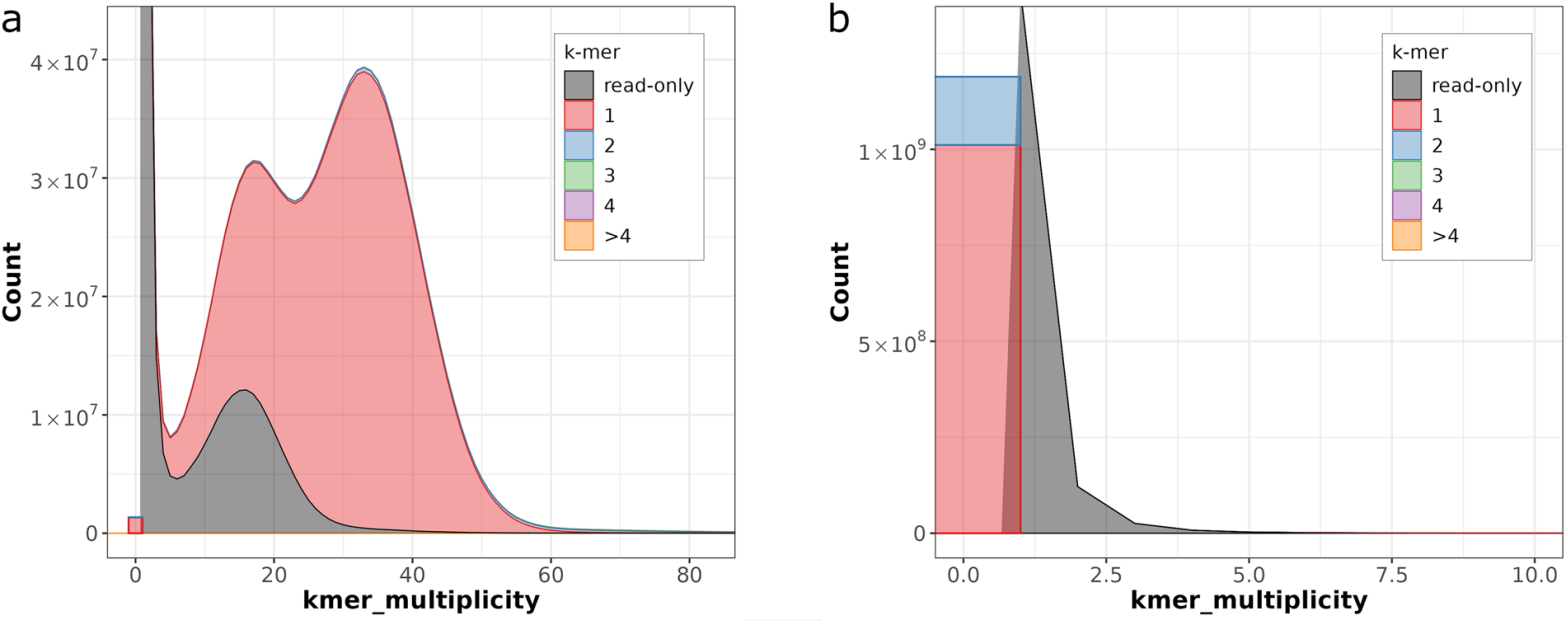
Copy number spectra generated by Merqury v1.3 from sand martin PacBio HiFi reads. Spectra were constructed using a 21-mer database prepared with Meryl v1.4.1. Colors indicate the number of times each k-mer is found in the genome assembly. The bar at the origin represents k-mers unique to the assembly (i.e. not supported by reads). (**a**) Spectrum of somatic k-mers. The read-only (grey) peak at approximately half the main coverage corresponds to heterozygous k-mers from the unrepresented haplotype. (**b**) Spectrum of testis-specific k-mers. The near-complete absence of these k-mers in the assembly confirms negligible GRC-derived contamination.

To assess the accuracy and completeness of the assembly, we performed k-mer-based validation. To avoid potential biases in the combined dataset introduced by removal of GRC reads, we extracted k-mers from somatic PacBio HiFi reads using meryl (k = 21), and compared them to the final assembly using Merqury v1.3^43^. The resulting spectra-cn plot is consistent with a pseudohaploid representation of the diploid genome (Fig. 5a). Most k-mers are present once, indicating minimal haplotig duplication. The estimated consensus quality value (QV) is 42.6, and k-mer completeness is 85.3%, consistent with expectations for a high-quality pseudohaploid assembly. To confirm that GRC-derived sequences are not represented in the assembly, we extracted testis-specific k-mers using the hapmers.sh script provided by Merqury and calculated their occurrence in the genome. Only 0.05% out of the 924,468 testis-specific k-mers were found in the assembly, supporting that it reliably reflects the somatic genome (Fig. 5b).

We assessed the completeness of the assembly by benchmarking with single-copy orthologs of *passeriformes_odb12* (2025-07-01) using BUSCO v5.8.2^24^ on genome mode. We found 6,487 complete single-copy genes, 35 complete duplicated genes, 31 fragmented genes, and 131 missing genes out of 6,684 BUSCO orthologs, indicating 97.6% genome completeness.

However, only two genes from the BUSCO gene set were found on the microchromosomes 16 and 30–37, commonly referred to as dot chromosomes. These chromosomes are known to be conserved throughout avian evolution and contain many highly expressed housekeeping genes^44^. The underrepresentation of the BUSCO genes on dot chromosomes is primarily attributed to the challenges in their assembling, which has led to a significant lack of dot chromosome gene annotations in OrthoDB^45^.

**Fig. 6:**
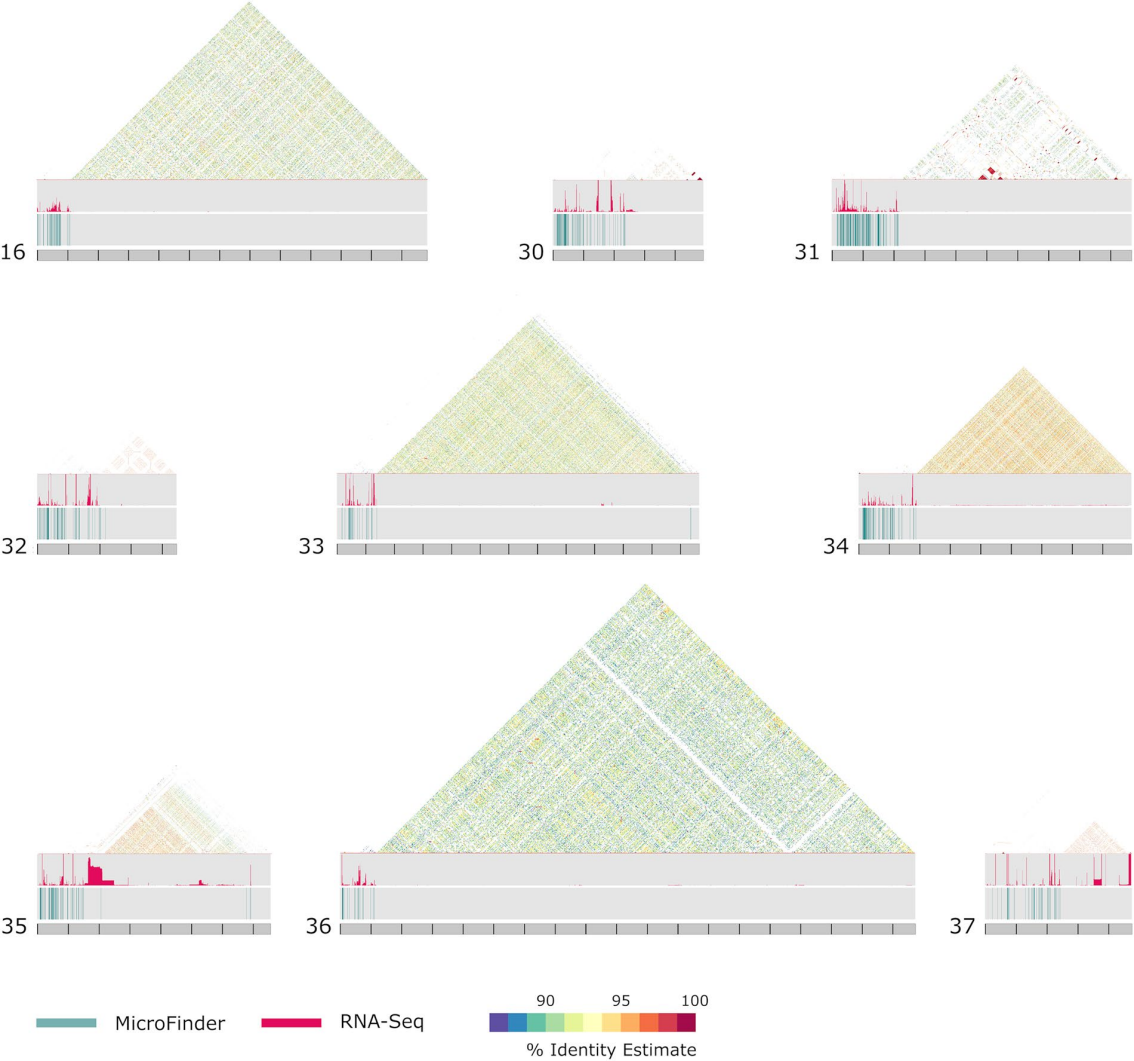
Structural and functional annotation of nine dot chromosomes in the sand martin genome assembly. For each chromosome, four panels are shown (from top to bottom): self-alignment dot plots representing repeat content; RNA-seq alignment counts from male sand martin somatic tissue mix in 10-kb windows (capped at 5,000 to emphasize low-coverage regions, red); distribution of MicroFinder protein set loci (blue); and chromosome schematics indicating 1-Mb intervals (black). Dot plots were generated with individual coordinate scales for each chromosome and then resized proportionally to reflect chromosome lengths. Color scale reflects the degree of sequence identity in the dot plots.

To assess the completeness of dot chromosome assemblies, we employed MicroFinder^46^, a recently developed pipeline that identifies conserved genes present in these regions by leveraging data from 11 high-quality, manually curated bird genomes. Of the 2,882 proteins in the MicroFinder set, 1,518 were found by the tool in the sand martin assembly with 1,308 of them located on chromosomes 16 and 30–37 (between 44 and 316 loci per chromosome) (Fig. 6). This indicates high assembly completeness and representation of dot chromosome gene content. MicroFinder loci tended to cluster in regions demonstrating high expression and low repeat density, as visualized in heatmap dot plots generated by ModDotPlot v0.9.1^47^ (Fig. 6). This is consistent with previous observations that identified gene-rich euchromatic regions and repetitive, gene-poor heterochromatic domains in dot chromosomes^48^.

## Data Availability

Raw sequencing data of *R. riparia*, including PacBio HiFi, Hi-C, and RNA-seq reads are available in the NCBI Sequence Read Archive (SRA) under accession number SRP610305^40^. The final chromosome-level genome assembly is available from NCBI under accession number GCA_055001455.1^41^. The transcriptome and genome annotation results are available via Figshare^42^.

## Code availability

Programs used in data processing were executed with default parameters unless otherwise specified in the Methods section. No custom code was employed for these analyses.
